# Novel insights into the pathogenesis of follicular lymphoma by molecular profiling of localized and systemic disease forms

**DOI:** 10.1038/s41375-023-01995-w

**Published:** 2023-08-10

**Authors:** Sabrina Kalmbach, Michael Grau, Myroslav Zapukhlyak, Ellen Leich, Vindi Jurinovic, Eva Hoster, Annette M. Staiger, Katrin S. Kurz, Oliver Weigert, Erik Gaitzsch, Verena Passerini, Marianne Engelhard, Klaus Herfarth, Klaus Beiske, Francesca Micci, Peter Möller, Heinz-Wolfram Bernd, Alfred C. Feller, Wolfram Klapper, Harald Stein, Martin-Leo Hansmann, Sylvia Hartmann, Martin Dreyling, Harald Holte, Georg Lenz, Andreas Rosenwald, German Ott, Heike Horn, German Lymphoma Alliance (GLA)

**Affiliations:** 1 Department of Clinical Pathology, Robert-Bosch Hospital, Stuttgart, Germany; 2 Dr. Margarete Fischer-Bosch Institute of Clinical Pharmacology, Stuttgart, Germany; 3 University of Tübingen, Tübingen, Germany; 4 Department of Medicine A, Department of Hematology, Oncology and Pneumology, University Hospital Münster, Münster, Germany; 5 Institute of Pathology, University of Würzburg and Comprehensive Cancer Center Main, Würzburg, Germany; 6 Department of Medicine III, University Hospital, LMU Munich, Munich, Germany; 7 Department for Radiotherapy, University Hospital of Essen, Essen, Germany; 8 Department of Radiation Oncology, University of Heidelberg, Heidelberg, Germany; 9 Department of Oncology, Oslo University Hospital, Oslo, Norway; 10 KG Jebsen center for B cell malignancies, Oslo, Norway; 11 Section for Cancer Cytogenetics, Oslo University Hospital, Oslo, Norway; 12 Institute of Pathology, University Hospital Ulm, Ulm, Germany; 13 Hematopathology, Lübeck, Germany; 14 Institute of Pathology, Hematopathology Section and Lymph Node Registry, University Hospital Schleswig-Holstein, Campus Kiel, Kiel, Germany; 15 Pathodiagnostik Berlin, Berlin, Germany; 16 Institute of Pathology, University Hospital Frankfurt, Frankfurt, Germany

**Keywords:** Cancer, Genetics

## Abstract

Knowledge on the pathogenesis of FL is mainly based on data derived from advanced/systemic stages of FL (sFL) and only small cohorts of localized FL (lFL) have been characterized intensively so far. Comprehensive analysis with profiling of somatic copy number alterations (SCNA) and whole exome sequencing (WES) was performed in 147 lFL and 122 sFL. Putative targets were analyzed for gene and protein expression. Overall, lFL and sFL, as well as *BCL2* translocation-positive (*BCL2*+) and –negative (*BCL2*−) FL showed overlapping features in SCNA and mutational profiles. Significant differences between lFL and sFL, however, were detected for SCNA frequencies, e.g., in 18q-gains (14% lFL vs. 36% sFL; *p* = 0.0003). Although rare in lFL, gains in 18q21 were associated with inferior progression-free survival (PFS). The mutational landscape of lFL and sFL included typical genetic lesions. However, *ARID1A* mutations were significantly more often detected in sFL (29%) compared to lFL (6%, *p* = 0.0001). In *BCL2* + FL mutations in *KMT2D*, *BCL2*, *ABL2*, *IGLL5* and *ARID1A* were enriched, while *STAT6* mutations more frequently occurred in *BCL2*- FL. Although the landscape of lFL and sFL showed overlapping features, molecular profiling revealed novel insights and identified gains in 18q21 as prognostic marker in lFL.

## Introduction

Follicular Lymphoma (FL) represents the majority of indolent B cell lymphomas accounting for 20% to 30% of all B cell lymphomas in the Western world with increasing numbers [1, 2]. The pathogenesis of FL is thought to involve repeated germinal center (GC) passages of B-cells with constitutive anti-apoptotic BCL2 expression induced by the hallmark t(14;18)(q32;q21) chromosome translocation. FL is considered an incurable disease, however, the clinical course varies significantly among patients [3, 4], and there is a wide range of therapeutic approaches [5]. The majority of FL are diagnosed in advanced stages (systemic FL, sFL), in contrast with only around 15% of FL being diagnosed in localized clinical stages (lFL) [6]. Various therapeutic options have been established aiming at curing localized stages by radiotherapy with or without combination with an anti-CD20 antibody (e.g., rituximab) and to prolong progression-free survival in systemic stages.

The biological mechanisms underlying the different clinical presentation and clinical course in FL have been in the focus of research for decades and advances in the genetic analysis of FL have shed light on the biological processes driving the pathogenesis and progression of FL. Our knowledge, however, is mainly based on data derived from analyses on sFL and only small cohorts of lFL have been characterized in-depth so far. One first hint pointing to relevant genetic differences between lFL and sFL was the finding of different frequencies of the founder *BCL2* translocation present in about 90% of sFL, but in only 50% of lFL, respectively [7, 8]. Moreover, gene expression (GE) profiling revealed different profiles of sFL and lFL [9]. Of importance, these data also revealed some particularities in a small subset of lFL that harbored a GE profile more closely resembling that of sFL. These cases clinically behaved more similar to sFL and had an inferior clinical outcome compared to the typical lFL [9]. Additional differences involve varying patterns of newly acquired N-glycosylation (N-glyc) sites between lFL and sFL [10].

In order to gain more detailed insights into the molecular make-up of lFL, comprehensive global analyses with SCNA (somatic copy number alteration) profiling and whole exome sequencing (WES) was performed in a large cohort of lFL. In addition, we compared the mutational profile as well as the SCNA landscape of lFL and sFL, and that of *BCL2* translocation-positive (*BCL2*+) and –negative (*BCL2*−) FL. By integrating information from published GE data sets [9], we generated a unique set of data enabling us to gain novel insights into the pathogenesis of both lFL and sFL.

## Material/subjects and methods

### Lymphoma specimens and study cohort

This study included optimally characterized FL mainly grades 1/2 and rare 3 A samples from different multicenter clinical trials and institutional archive collections. All samples were diagnosed by expert hematopathologists according to the guidelines of the updated 4th edition of the World Health Organization classification of tumors of haematopoietic and lymphoid tissues [1]. Clinical staging informed on localized stages I, II and IIIA (lFL), as well as systemic stages III and IV (sFL). The majority of localized stage FL tumor samples was collected from prospective randomized trials within the German Lymphoma Alliance [GLA, former: German Low Grade Lymphoma Study Group (GLSG)], enrolling patients having received different radiotherapy treatments [11, 12]. In addition, localized-stage FL tumor samples from the institutional archives of the Robert–Bosch–Krankenhaus, Stuttgart, Germany and Oslo University Hospital, Oslo, Norway, with different treatment modalities were available. The systemic-stage FL tumor samples were collected from the GLSG2000 study [13] and from the Robert–Bosch–Krankenhaus Stuttgart, Germany. All trials were conducted in accordance with the Helsinki Declaration. The protocols had been approved by the ethics review committee of each participating center, as had been done also for the patient samples outside clinical trials. Nucleic acid extraction and quality control (QC) assessment are described in the Supplementary Information.

### WES and OncoScan CNV Assay for the detection of somatic mutations and SCNA

For WES 164 samples were analyzed (140 lFL vs. 24 sFL), of which 22 had matched normal samples and 142 were unpaired. In addition, we included 35 normal samples from healthy donors [14]. Together with the 22 paired germline samples, they formed the panel of normals (PON) used for variant filtering (*n* = 57). To profile SCNA, 149 tumor samples from lFL (132 FFPE and 15 fresh frozen) and 122 unpaired samples from sFL were measured. Exome sequencing and Oncoscan CNV Assay (Thermo Fisher Scientific, Waltham, Massachusetts, USA) were performed as described in the Supplementary Information.

### Fluorescence in-situ hybridization (FISH), real-time PCR (RT-PCR) and immunohistochemical analyses

For the majority of lFL and sFL *BCL2* translocation status had already been published [7, 8]. For the remaining tumor specimens without *BCL2* translocation status, FISH and delta-PCR were used to evaluate the *BCL2* break status of FL specimens. *BCL6* translocation status was assessed in *BCL2-* FL. For the validation of the novel, recurrent SCNA FISH was performed. Gene expression of distinct target genes in selected samples was analyzed using TaqMan probes and an AB gene expression master mix (all reported in the Supplementary Information). For immunohistochemical staining of IKZF1, the IKZF1 antibody (clone D6N9Y, pH 9.0, 1:500) was used (Rabbit mAb #14859, Cell Signaling, Leiden, Netherlands). Nuclear IKZF1 staining in lymphocytes was recorded as low and high expression.

### Clinical correlations

Clinical outcome was measured by the time to event data progression-free survival (PFS) from treatment start to stable disease, progression or death from any cause. PFS was censored at the latest tumor assessment data when no progression or death had been reported. For statistical evaluation of the prognostic value of genetic aberrations in lFL time to event variables were analysed with Cox proportional hazards regression, and the Wald test *P*-values for regression coefficients were reported. The *P*-values indicated in the Kaplan–Meier plots were calculated with the log-rank test. The *P*-values were not adjusted for multiple testing, as the results were interpreted in a purely hypothesis-generating and explorative way.

## Results

Altogether, 269 FL samples were available for SCNA profiling including 147 lFL and 122 sFL. Somatic mutations were analyzed in 164 specimens comprising 140 lFL and 24 sFL (Supplementary Fig. S1). Among lFL, 90 samples had clinical stage I and 46 cases clinical stage II. SCNA and WES data were compared between lFL (stages I and II) and sFL, as well as between *BCL2*+ and *BCL2*− FL.

### WES and SCNA profiling reveals novel overlapping features in the molecular landscape of lFL and sFL

The mean variant count per sample and Mb was 1.35 for the entire cohort and 1.36 for lFL and 1.53 for sFL. Comparison with other cancer entities from TCGA suggests a moderate tumor mutational burden (TMB; see Supplementary Fig. S2A). lFL harbored an average number of 80.1 mutations per sample, slightly lower than the 90.6 mutations per sample in sFL. The number of mutations per sample did not differ significantly between lFL and sFL (Supplementary Fig. S2B).

**Fig. 1:**
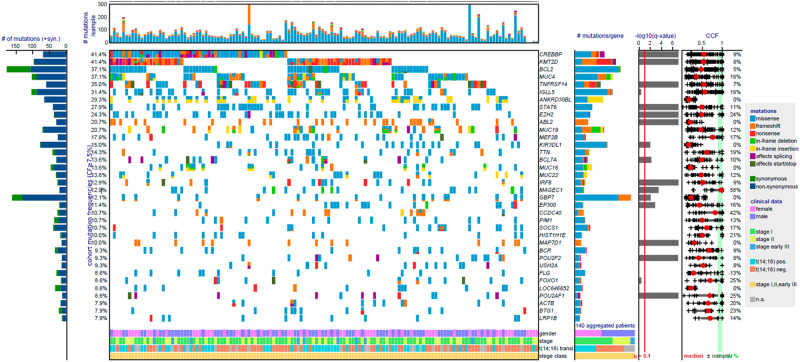
Mutational landscape in lFL. All called non-synonymous and synonymous mutations in significant genes according to MutSig2CV v3.11 (qM2CV < 0.1, cohort frequency ≥ 10% in lFL), but also non-significant biologically-relevant genes (e.g., *BCL2*) are color-coded and shown for sample per column, ranked by cohort frequency. Samples are ordered by waterfall sorting based on binary gene mutation status. The bar graph on the left shows the ratio of non-synonymous (blue) and synonymous (green) mutations per gene. At the top, the tumor mutational burden (TMB) per sample (mutations/sample/Mb) is depicted. On the right, occurring types of mutation, q values (M2CV) and cancer cell fractions (CCF) are shown per gene.

The mutation patterns of lFL stages I and II did not show any difference (Supplementary Table S3). The most frequently (≥10%) mutated driver genes in lFL, as defined by MutSig2CV (Supplementary Table S4), were *CREBBP* and *KMT2D* (41% each), *TNFRSF14* (35%), *STAT6* (28%), *EZH2* (23%), *ABL2* (21%), *KIR3DL1* (14%), *BCL7A* (14%), *IRF8* (13%), *MAGEC1* (13%), *GBP7* (12%) and *EP300* (11%). A high frequency of mutations was also identified in *BCL2* (37%), however, about 50% of them were synonymous (Fig. 1, Supplementary Table S5). A similar mutation profile was observed in sFL, delineating most frequent driver mutations in *KMT2D* (67%), *CREBBP* (38%) and *TNFRSF14* (33%). Similar to lFL, 50% of mutations encountered in *BCL2* represented synonymous mutations. In addition, *ARID1A* (29%), *ABL2* (25%), *EZH2* (21%) *IRF8* (21%) *STAT6* (21%), *MAP7D1* and *POU2F2* (17% each) genes were frequently mutated (Supplementary Fig. S3 and Supplementary Table S5).

The high mutation rate affecting *BCL2* might be caused by aberrant somatic hypermutation (SHM). However, apart from *BCL2* and *PIM1*, none of typically SHM-affected genes (*BCL6*, *PIM1*, *MYC*, *RHOH*, *PAX5* and *CD95*) [15] was targeted by SHM in the present study. *BCL2* and *PIM1* were frequently mutated in both lFL and sFL, with equal number of mutations per sample (*BCL2*: 3.1 mutations/sample in lFL and 2.6 mutations/sample in sFL; *PIM1*: 3 mutations/sample in both). Samples with high number of *BCL2* mutations were more frequently observed in the lFL stage I cohort (15/24, 63% vs. 9/24, 37% in lFL stage II; *p* < 0.01), while no difference was detected in *PIM1*-mutated samples. Tumor samples with an increased *BCL2* mutation rate were enriched in tumor samples with *BCL2* translocation (25/33, 76% vs. 8/33, 24% without *BCL2* translocation; *p* < 0.01).

1468 SCNA in lFL and 1252 SCNA in sFL were identified (Supplementary Tables S6 and S7). The average number of SCNA per case was 10 (range: 0–54) for lFL and 10.3 for sFL (range: 1–39). A similar pattern of frequent alterations was observed in FL stages I and II. FLI samples harbored an average of 9.26 SCNA per sample and FLII 11.36. Differences were restricted to few regions without reaching significance (Supplementary Fig. S4).

**Fig. 2:**
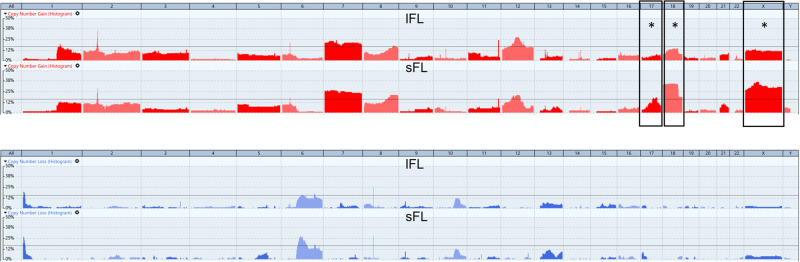
Comparative analysis of somatic copy number alterations (SCNA) in FL. Frequency of SCNA in the entire cohort of localized FL (lFL) and systemic FL (sFL). Copy number gains along the genome are depicted in red (above); copy number losses are illustrated in blue (below). The dashed line indicates the threshold for recurrent SCNA ≥ 15%. Fisher’s exact test and Benjamini–Hochberg correction for multiple testing was applied to determine significant differences (*q* < 0.05) in the SCNA frequency of lFL and sFL. Significant differences between lFL and sFL are marked with a black frame and asterisk.

As for the mutational profile, the landscape of SCNA in lFL and sFL did not show major differences. Recurrent SCNA with a frequency of ≥15% were observed in both lFL and sFL in regions previously described to be frequently altered in FL [16–19]. Those included gains occurring in the X chromosome and in 1q21, 2p16, 7, 8q24, 12q, and 18q, as well as losses in 1p36 and 6q (Fig. 2), albeit with differing subregions affected and with different frequencies in lFL and sFL (Table 1).

**Table 1:** Recurrent somatic copy number alterations (SCNA) ≥ 15% in localized (lFL) and systemic FL (sFL).

	lFL (*n* = 147)	sFL (*n* = 122)
Gains		
2p16	48 (33%)	34 (28%)
7	38 (26%)	34 (28%)
8q24	24 (16%)	25 (21%)
11q24	32 (22%)	20 (16%)
12q	44 (30%)	30 (25%)
17q21	9 (6%)	23 (19%)
18	21 (14%)	44 (36%)
X	24 (16%)	47 (39%)
Losses		
1p36	24 (16%)	31 (25%)
6q12	15 (10%)	24 (20%)
6q23	22 (15%)	21 (17%)
8p11	32 (22%)	32 (26%)

**Fig. 3:**
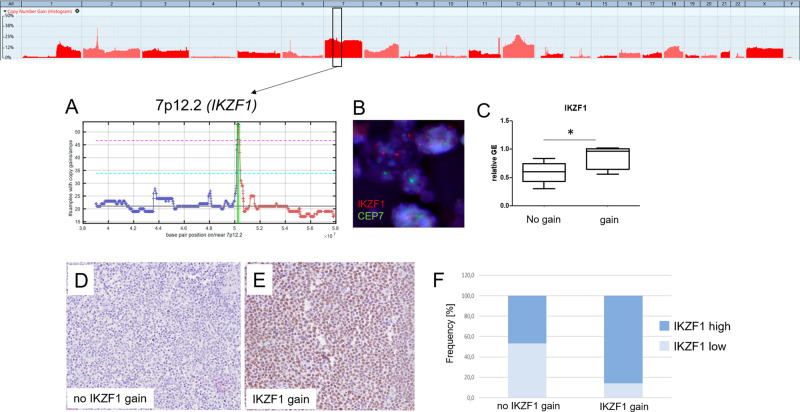
Identification of *IKZF1* as significant alterated gene in 7p12.2 by GISTIC. Chromosome 7 was affected by wide whole-arm gains. Applying the GISTIC algorithm enabled the identification of one single gene in chromosome 7p12.2, significantly (FDR *q* < 0.1) gained in lFL and sFL. Chromosomal gains of *IKZF1* in 7p12.2 (**A**) was validated with locus-specific probes by fluorescence in situ hybridization (**B**). mRNA expression of *IKZF1* (**C**) was significantly increased in FL samples with gains in 7p12.2 as measured by Mann–Whitney *U*-test. *IKZF1* protein expression in tumor samples without (**D**) and with *IKZF1* gain (**E**). An increased *IKZF1* protein expression was observed in samples with *IKZF1* gain, but to a lesser extent were also present in samples without *IKZF1* gain (**F**).

Apart from these known and FL-typical alterations, hitherto unknown novel recurrent focal SCNA were detected both in lFL and sFL, as well as significant regions of gains identified by the GISTIC algorithm (Supplementary Fig. S5). The SCNA comprised focal losses in 8p11.22 including the metallopeptidase genes *ADAM18* and *ADAM32* in 22% of lFL (*q* = 1.4E-15) and focal gains affecting 11q24.3 harboring cancer-associated genes *ETS1* and *FLI1* in 22% of lFL (*q* = 2E-27). Moreover, the *FCRL5* gene mapping in 1q23.1 (*q* = 6.8E-35) and the *IKZF1* gene in 7p12.2 (*q* = 7,9E-37) were identified to be significantly gained in 33% and 35% lFL, respectively (Fig. 3A, Supplementary Fig. S6 and Table S7). Those significant regions were observed to be altered also in sFL with similar frequencies (*ETS1*/*FLI1*: 29%, *FCRL5*: 20% and *IKZF1*: 30%). Loss of *ADAM32* as well as gains in *ETS1*, *FCRL5* and *IKZF1* were validated by FISH (Fig. 3B and Supplementary Fig. S6). The mRNA expression of *ADAM32* was significantly reduced in samples with 8p11.22 loss (*p* = 0.0103) compared to samples without 8p11.22 deletion. For regions of chromosomal gains, *ETS1 and FCRL5* expression only showed a trend towards higher expression in lFL with gains in 11q24.3 and 1q23.1, respectively. (Supplementary Fig. S6). In contrast, expression of IKZF1 was significantly upregulated in cases with *IKZF1* gain compared with samples lacking the gain (*p* = 0.0307) (Fig. 3C). In addition, gains in the *IKZF1* gene correlated with high expression of the IKZF1 protein, although a significant proportion of samples without *IKZF1* gain also showed enhanced IKZF1 protein expression (Fig. 3D–F).

### Differences in the SCNA and mutation pattern between lFL and sFL

Despite highly similar overall SCNA and mutational profiles in both lFL and sFL, some significant differences were observed.

Gains of 17q21 were found in 6% (9/147) lFL but in 19% (23/122) of sFL (*q* = 0.0084). Moreover, X-chromosomal gains were detected in 16% (24/147) lFL and in 39% (47/122) sFL (*q* = 0.000576). Chromosome 18 was affected by gains in 14% (21/147) of lFL and in 36% (44/122) sFL (*q* = 0.0003, Fig. 2). The most significant regions in lFL as determined with the GISTIC algorithm were 18q21.32 and 18q21.33, where the *MALT1* and *BCL2* genes are located (*q* = 8.1E-15 and *q* = 0.0005). Moreover, deletions in chromosome 6q12-q21 were frequently observed in sFL (29% vs. 14% in lFL, n.s.).

**Fig. 4:**
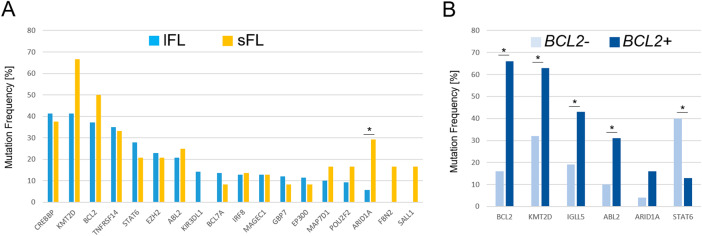
Comparative mutational profiles in FL. Most frequently mutated genes in lFL and sFL, indicating a significant difference (*) in *ARID1A* mutations in lFL and sFL (**A**). Wilcoxon rank sum test, followed by Benjamini–Hochberg correction for multiple hypothesis testing, were used to determine significant differences between lFL and sFL (*q* < 0.1). Comparing the mutation frequency in *BCL2* translocation-negative (*BLC2*-) and –positive (*BCL2*+) FL revealed significant differences in mutation frequencies of *BCL2*, *KMT2D*, *IGLL5* and *ABL2* enriched in *BCL2*+, while *STAT6* mutations more frequently occurred in *BCL2*− (**B**).

Comparison of the mutational patterns of lFL and sFL revealed differences in the frequency of mutations affecting *ARID1A* and *KMT2D*: for both genes, higher mutational frequencies were seen in sFL (*ARID1A*: 29% vs. 6% in lFL, *q* = 0.0041; *KMT2D*: 67% vs. 41% in lFL, n.s., Supplementary Table S3). Although mutations in the *CREBBP* and *KMT2D* genes were observed with the highest frequency in both lFL and sFL, differences in the mutational spectrum were observed. In lFL, both genes harbored splicing site mutations, whereas no such mutations were detected in sFL (Supplementary Fig. S7A, B). In addition, *KIR3DL1* mutations were exclusively found in lFL (15%, Fig. 4A). *KIR3DL1* variants were predominantly missense mutations and occurred exclusively at the known hotspot residues in the immunoglobulin domain (Supplementary Fig. S7C). When comparing GE data [9] from patients with and without *KIR3DL1* mutations (*n* = 44 vs. *n* = 8), reduced mRNA expression was observed for the *CD4*, *ITK* and *SH2D1A* genes in *KIR3DL1* mutated lFL (*p* = 0.036, *p* = 0.037 and *p* = 0.045, respectively, Supplementary Fig. S7D), all involved in NK cell activation [20–22].

### The majority of *BCL2*+ and *BCL2*- FL harbor overlapping SCNA profiles

It is well established that the majority of lFL lack *BCL2*-translocations [7]. In the present cohort, *BCL2* translocations were observed in 49% of lFL (66/140) and in 92% of sFL (70/76). Subsequently, we analyzed the distribution of SCNAs for *BCL2* + (*n* = 66) and *BCL2*− (*n* = 68) lFL. Of pivotal importance, the overall SCNA pattern for *BCL2*+ and *BCL2*- FL resembled the overall cohort without significant differences. In contrast to previous studies of sFL [23, 24], in lFL, 18q21 gains including the genes *BCL2* and *MALT1* were not restricted to *BCL2* + FL, but to an equal percentage also occurred in *BCL2-* FL (Supplementary Fig. S8).

Los-de Vries et al. recently reported that *BCL2-* lFL can be distinguished from *BCL2*- sFL with regard to their underlying *CREBBP* and *STAT6* mutation patterns, as well as to their *BCL6* translocation status [25]. In our cohort, presence of *BCL6* rearrangements was tested in *BCL2*- lFL (*n* = 53) and *BCL2*- sFL (*n* = 52), revealing rearrangements in 17% lFL (9/53, *n* = 4 stage I, *n* = 5 stage II) and 19% sFL (10/54, n.s.). *CREBBP* and *STAT6* mutations were more frequently encountered in *BCL2*- lFL (*CREBBP*: 41% vs. 29%; *STAT6*: 35% vs. 14% in *BCL2*- sFL; Supplementary Fig. S9), but the difference was not statistically significant.

### Significant differences in the mutational landscapes of *BCL2*+ and *BCL2*- FL

In contrast to the overall similar mutational profiles between sFL and lFL, the evaluation of mutations in *BCL2+* and *BCL2-* FL revealed significant differences in the frequencies of alterations in *KMT2D, BCL2*, *ABL2, IGLL5* and *STAT6* (Supplementary Table S3). *KMT2D* mutations were found in 63% of *BCL2* + FL and 32% *BCL2*- FL (*q* = 0.0011). The same applied to *BCL2* mutations which occurred more frequently in *BCL2* + FL (66% vs. 16% *BCL2*- FL, *q* = 1.2E-07). In addition, *BCL2* + FL harbored significantly more mutations in *ABL2* (31% vs. 10%, *q* = 0.0084) and *IGLL5* (43% vs. 19%, *q* = 0.0084). Although not significant, mutations in *ARID1A* occurred more frequently in *BCL2* + FL (16% vs. 4%, *q* = 0.0584). In contrast, mutations in *STAT6* were more frequently detected in 40% of *BCL2-* FL but in only 13% of *BCL2* + FL (*q* = 0.0095, Fig. 4B).

**Fig. 5:**
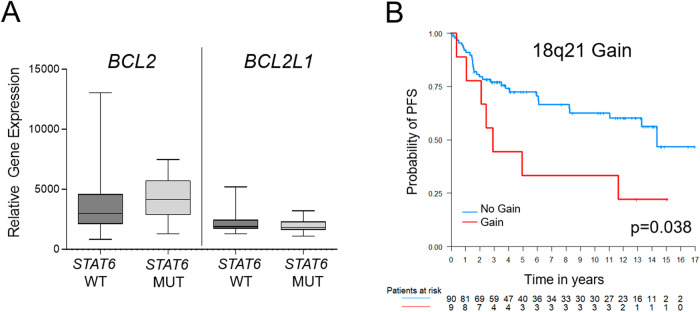
*STAT6* mutations and 18q gains in *BCL2* translocation-negative lFL. mRNA expression of the anti-apoptotic genes *BCL2* and *BCL2L1* in relationship to the underlying *STAT6* mutation status in *BCL2* translocation-negative (*BCL2*-) lFL did not show any differences in *STAT6* wildtype (WT) or mutant (MUT) samples (**A**). Gains in chromosome 18q21 (including the *BCL2* locus) were associated with decreased progression-free survival (PFS) in the patient cohort of lFL as illustrated by Kaplan–Meier plot (**B**). Time to event variables were analysed with Cox proportional hazards regression. The p*-*values indicated in the Kaplan–Meier plots were calculated with the log-rank test.

*STAT6* has been described to be involved in the regulation of apoptosis by mediating upregulation of *BCL2* and the anti-apoptotic *BCL2L1* (*BCL*-*XL*) gene [26], thus possibly providing an alternative mechanism for the deregulation of BCL2 in FL samples without *BCL2* translocation. We thus analyzed the gene expression of *BCL2L1* in *STAT6* mutant vs. *STAT6* wildtype samples. However, *BCL2L1* expression did not differ significantly in *BCL2*- lFL with or without *STAT6* mutations (Fig. 5A).

### Gains in 18q21 have prognostic impact in lFL

Although the frequency of *BCL2* translocation is significantly lower in lFL, gains of chromosomal material from 18q affecting the *BCL2* locus and thus providing an alternative mechanism for *BCL2* deregulation, more frequently occurred in sFL (Fig. 2). The majority of lFL with 18q21 gains was negative for the *BCL2* translocation (*n* = 10 with FISH data; 7/10, 70%). Of those, however, all showed increased expression of the BCL2 protein. Of interest, in univariate analysis, an 18q21 gain was significantly associated with inferior PFS in patients with lFL (*p* = 0.038, Fig. 5B). Other gene mutations (*CREBBP*, *KMT2D*, *BCL2*, *STAT6*, *ARID1A*, *ABL2*) or SCNA tested (7p12 and 11q24 gains, losses in 8p11, *BCL2* translocations) did not show any association with clinical outcome in patients with lFL.

## Discussion

Previously published data suggested that there are some molecular differences between lFL and sFL such as a lower frequency of *BCL2* translocations in lFL, varying gene expression profiles and differences in features related to the microenvironment [8–10, 23, 27]. However, data derived from lFL are still sparse [25], and no comprehensive data set on whole exome sequencing was available so far. One essential and unmet issue is the question of biological drivers in lFL lacking the *BCL2* translocation. We thus initiated a comprehensive in-depth molecular profiling study of a large cohort of lFL using global SCNA and WES profiling. The first interesting finding of our study was that the SCNA and mutational landscapes of lFL and sFL are highly similar. In contrast to previous findings describing an increasing genomic complexity in higher stages of FL [28] the average number of SCNA and mutations per sample did not differ significantly between lFL stage I, lFL stage II and sFL. These largely overlapping biological features of lFL and sFL are surprising given the tremendously differing prognostic impact of a diagnosis of lFL versus sFL. Our data thus confirm and extend recently published data on lFL versus sFL that already indicated a close genetic relationship of lFL and sFL with overlapping features on SCNA and mutational level [25]. This is in clear contrast, however, to the differences observed between lFL and sFL on the RNA and microenvironmental levels [9, 27], suggesting alternative mechanisms to be important in lFL and sFL, e.g., epigenetic alterations.

Although extensive analyses of SCNA have been performed in FL, the segregation of driver and passenger genes has not been comprehensively determined until now. Thus, some of the hitherto recognized major players have been identified in chromosomal regions frequently harboring losses or gains (e.g., *PTEN* in 10q, *TNFRSF14* in 1p36), but some recurring alterations (e.g., gains in chromosome 7 and 17) suggest additional, yet unidentified, potential drivers. SCNA may simultaneously affect up to thousands of genes, while selective benefits of driver alterations are likely to be mediated by only one or a few of these genes. With the application of the GISTIC algorithm [29], assuming that chromosomal regions containing driver events should be altered at higher frequencies than regions containing only passengers [30–33], novel target regions were identified, among others in the region of gain in 7p12.2 affecting lFL and sFL.

Moreover, gains of chromosomal band 7p12.2 turned out to be highly significant in lFL and sFL. With the GISTIC algorithm, the focal region of gain, containing exclusively the *IKZF1* gene, was identified in 35% of lFL and 30% of sFL. This zinc finger DNA-binding protein is of special interest due to its involvement in chromatin remodeling processes during B-cell differentiation: IKZF1 interacts with IRF4 and a positive coactivator (PC4) to orchestrate terminal differentiation into plasma cells [34]. Intriguingly, knock-down of IKZF1 sensitizes tumor cells in DLBCL to treatment with the EZH2 inhibitor tazemetostat [35], not only emphasizing its biological, but also a possible clinical impact in lymphoma.

Apart from these novel insights into the SCNA landscape of lFL, to the best of our knowledge, this is the first study performing WES in lFL. The mutation-based profiles of lFL and sFL were found to be remarkably similar corroborating previous findings in targeted sequencing approaches [25, 36], as were the median numbers of mutations in each type. With only targeted NGS analyses of lFL available until now, a global view towards the landscape of mutations in lFL and sFL has not yet been provided. Consequently, in the present study, not only differences in the frequency of known mutations were observed between lFL and sFL, but for the first time also *KIR3DL1* gene mutations selectively occurring in lFL (15%). The *KIR3DL1* gene is part of the *KIR* gene cluster, encoding killer-cell immunoglobulin-like receptors. These transmembrane glycoproteins are involved in the modulation of the NK cell response [37, 38]. Interaction of KIRs and their ligands mediate NK cell activation and have been reported to influence the therapy response of FL treated with rituximab [39] which fits well into the concept that a reduced proportion of NK cells may negatively impact clinical outcome of FL patients [40]. Of special interest, patients with *KIR3DL1* mutations show significantly reduced expression of NK cell activation markers *CD4* [20], *ITK* [21] and *SH2D1A* [22] thus emphasizing the potential impact of *KIR3DL1* mutations in the modulation of the microenvironment in lFL.

*KMT2D* and *ARID1A*, modulators of chromatin remodeling processes, are more frequently mutated in sFL than in lFL, as previously published [25], possibly contributing towards altered chromatin regulation in lFL and sFL. In contrast, mutations in *CREBBP* were equally distributed in lFL and sFL [25]. Although *KMT2D* and *CREBBP* were overall shown to be the most frequently mutated genes both in lFL and sFL in the present study, a different pattern of mutations was observed, including splicing site mutations in both genes exclusively occurring in lFL. Splicing site mutations can result in either complete skipping of the exon or the retention of an intron, possibly leading to altered gene expression and the generation of truncated proteins [41], again pointing to differential chromatin remodeling processes in lFL and sFL. The functional consequences of *CREBBP* and *KMT2D* splicing site mutations in lFL, as well as their effects on gene and protein expression, however, need to be determined.

Although the lower frequency of *BCL2* translocations is considered a hallmark feature of lFL, the majority of these FL nevertheless express the BCL2 protein [7]. Gains in 18q21 can be observed in *BCL2*- FL with BCL2 protein expression [24], thus suggesting 18q21-gains as a surrogate for *BCL2* translocation. In the present study, gains of the *BCL2* locus were significantly enriched in sFL, however, of note, these 18q21 gains were accumulated in *BCL2−* FL. This is in contrast to previous findings of sFL, describing an occurrence of 18q21 gains predominantly in *BCL2* + FL and only rarely occurring in *BCL2*− FL [23]. While presence or absence of *BCL2* translocations did not predict the clinical outcome of patients with FL, gains/amplification of 18q21 correlated with an inferior overall survival in sFL [23]. Of special interest, this was also evident in the present cohort of lFL, where gains in 18q21 were associated with reduced PFS. The majority of lFL with 18q21 gains was negative for the *BCL2* translocation (*n* = 10 with FISH data; 7/10, 70%). Moreover, samples with gains from 18q21 were predominantly associated with concomitant gains of *BCL2* and *MALT1* genes (16/22, 73%). Of interest, in DLBCL, 18q21 gains (including *BCL2* and *MALT1*) are enriched in the ABC subtype and are associated with poorer clinical outcome [42]. Our finding of reduced PFS in *BCL2* - lFL patients with 18q21 gains might possibly bridge the finding that transformed FL lacking a *BCL2* translocation often are of ABC subtype [43].

In conclusion, our data generated from a large scale genetic approach reveal a surprisingly large overlap in the genetic landscape of lFL and sFL. Notwithstanding this, there are some striking differences between lFL and sFL in the frequency of SNCA and mutations, in particular with regard to the underlying *BCL2* translocation status. The enrichment of *ARID1A* and *KMT2D* mutations in *BCL2* + FL, independent of the clinical stage, might indicate an altered accessibility to the chromatin structure of the tumor cells. This, together with the functional consequences of splicing site mutations in *CREBBP* and *KMT2D*, exclusively occurring in lFL, needs to be determined. The finding of *KIR3DL1* mutations solely in lFL emphasizes the significant impact provided by the microenvironment as had been already reported [44]. Although *BCL2*+ and *BCL2*− FL do not differ in their prognosis neither in limited nor in systemic stages, we were able to show that gains in chromosome 18q21, enriched in *BCL2*− lFL, are associated with an inferior PFS in patients with lFL. This might prove valuable in the risk stratification of patients at diagnosis and could contribute to an optimized risk-adapted therapy of lFL.

### Supplementary information

Supplementary Information

Supplementary Table S1

Supplementary Table S2

Supplementary Table S3

Supplementary Table S4

Supplementary Table S5

Supplementary Table S6

Supplementary Table S7

## Data Availability

The WES and SCNA data generated in this study have been deposited in the European Genome-phenome Archive (EGA) under study accession EGAS00001006927.
