# Supplementary material for: Novel insights into the pathogenesis of follicular lymphoma by molecular profiling of localized and systemic disease forms
Source: Leukemia. 2023 Aug 10;37(10):2058–65. doi: 10.1038/s41375-023-01995-w (PMC10539171; doi:10.1038/s41375-023-01995-w)
Supplement: Supplementary file 1 — Supplementary Information [file 41375_2023_1995_MOESM1_ESM.docx]

**Supplementary Information**

**Novel insights into the pathogenesis of follicular lymphoma by molecular profiling of localized and systemic disease forms**

Sabrina Kalmbach^1,2,3#^, Michael Grau^4#^, Myroslav Zapukhlyak^4^, Ellen Leich^5^, Vindi Jurinovic^6^, Eva Hoster^6^, Annette M. Staiger^1,2,3^, Katrin S. Kurz^1^, Oliver Weigert^6^, Erik Gaitzsch^6^, Verena Passerini^6^, Marianne Engelhard^7^, Klaus Herfarth^8^, Klaus Beiske^9^, Francesca Micci^10^, Peter Möller^11^, Heinz-Wolfram Bernd^12^, Alfred C. Feller^12^, Wolfram Klapper^13^, Harald Stein^14^, Martin-Leo Hansmann^15^, Sylvia Hartmann^15^, Martin Dreyling^6^, Harald Holte^9^, Georg Lenz^4^, Andreas Rosenwald^5^, German Ott^1,2^, Heike Horn^1,2,3^ , German Lymphoma Alliance (GLA)*

^1^Department of Clinical Pathology, Robert-Bosch Hospital, Stuttgart, Germany

^2^Dr. Margarete Fischer-Bosch Institute of Clinical Pharmacology, Stuttgart, Germany,

^3^University of Tübingen, Tübingen, Germany,

^4^Department of Medicine A, Department of Hematology, Oncology and Pneumology, University Hospital Münster, Münster, Germany,

^5^Institute of Pathology, University of Würzburg and Comprehensive Cancer Center Main, Würzburg, Germany,

^6^Department of Medicine III, University Hospital, LMU Munich, Munich, Germany

^7^Department for Radiotherapy, University Hospital of Essen, Essen, Germany,

^8^Department of Radiation Oncology, University of Heidelberg, Heidelberg, Germany,

^9^Department of Oncology, Oslo University Hospital, Oslo, Norway and KG Jebsen center for B cell malignancies,

^10^Section for Cancer Cytogenetics, Oslo University Hospital, Oslo, Norway.

^11^Institute of Pathology, University Hospital Ulm, Ulm, Germany

^12^Hematopathology, Lübeck, Germany,

^13^Institute of Pathology, Hematopathology Section and Lymph Node Registry, University Hospital Schleswig-Holstein, Campus Kiel, Kiel, Germany,

^14^Pathodiagnostik Berlin, Berlin, Germany,

^15^Institute of Pathology, University Hospital Frankfurt, Frankfurt, Germany,

# These authors contributed equally to this work

* A list of authors and their affiliations appears at the end of the paper

**Supplementary Methods**

Sample Preparation and quality control

Samples from the different study cohorts were available as either formalin-fixed paraffin-embedded (FFPE) or fresh-frozen (FF) tissues.

A total of 155 lFL samples were included into the study based upon sufficient quality for subsequent analyses. SCNA data were obtained from 147 lFL and a comparative cohort of 122 sFL. WES was performed from 140 lFL and a comparative cohort of 24 sFL. Combined SCNA and WES data were available from 132 lFL (Supplementary Figure S1, Supplementary Table S1). GE profiling data have been previously reported (1). GE data of 184 genes were available from 52 lFL samples.

Nucleic acid extraction was performed using the AllPrep DNA/RNA FFPE Kit (Qiagen, Hilden, Germany). FF samples and cell lines were treated using the AllPrep DNA/RNA Mini Kit (Qiagen, Hilden, Germany), according to the manufacturer’s protocol. DNA concentrations were determined using the Qubit DNA Quantification Assay (Qiagen, Hilden, Germany). Assessment of DNA quality for subsequent SCNA analysis was performed by quantitative real-time PCR on an Applied Biosystems 7500 instrument (Thermo Fisher Scientific, Waltham, Massachusetts, USA) using a TaqMan Gene Expression Assay (Thermo Fisher Scientific, Waltham, Massachusetts, USA) for the *FTH1* gene (Hs01694011_s1). Quality PCR for each DNA targeting the *HBB* gene was accomplished as previously described (2). Quality assessment for exome sequencing was performed using the Illumina Infinium QC FFPE Kit (Illumina, San Diego, California, USA) was conducted according to manufacturer’s protocol.

Sequencing and preprocessing

For 164 tumor and 57 germline samples, whole exome sequencing was performed by capturing the exome by the SureSelect Human All Exon V6 Kit (Agilent, Santa Clara, California, USA). GATC Biotech (AG, Konstanz, Germany, now part of Eurofins Scientific SE, Luxembourg City, Luxembourg) utilized an Illumina HiSeq platform for 25 tumor and 17 germline frozen samples (human all exon enrichment, Illumina HiSeq 4000, SureSelect All Exon V6). For the remaining samples, NOVOGENE (Co., Ltd., Beijing, China) utilized Illumina NovaSeq sequencers. Sequenced reads were preprocessed and quality-controlled using Cutadapt (3), Trim Galore! (4), and FastQC (5). No sample was excluded by low-level QC. WES filtering hierarchy is described in Supplementary Table S2.

WES Data analysis

*Sequence alignment, quality control, effective coverage*. We used HISAT2 v2.2.1 (6) to align measured sequence reads against the current human reference genome from the Genome Reference Consortium (GRCh38) (7). Supplementary Table S1 summarizes HISAT alignment rates and resulting read counts for each sample. We checked concordance between tumor and normal samples using Conpair v0.2 (8) and NGSCheckMate v1.0.0 (9), confirming that each tumor/normal pair had a common germline.

*Variant discovery*. For variant discovery based on aligned reads, we utilized the Genome Analysis Toolkit v4.1.2.0 (GATK) (10). After base quality score recalibration, we utilized Mutect2 from this GATK for variant discovery. To quantify effective coverage, only reads aligned by HISAT2 that also passed GATK and Mutect2 read level quality control read filters were used for variant detection (the resulting distribution of effective coverage is shown in Supplementary Figure S2). No samples were excluded due to insufficient effective coverage.

*Basic variant filtering.* For basic variant filtering we first performed variant discovery with the same experimental and analytical pipeline also for the 57 normal controls (comprised of 22 paired normals from patients and additional 35 germline samples from healthy donors) to create a panel of normal variants (PON). A variant was included in the PON if Mutect2 confirmed its significance relative to the reference genome in at least two independent subjects. This PON was subsequently used as one step to filter germline variants and potential pipeline-specific artifacts when applying Mutect2 in somatic variant discovery mode for the tumor samples. Additionally, we utilized the gnomAD database as a large population germline resource based on the Exome Aggregation Consortium ExAC (11). For tumor samples having matched normal samples, we utilized the more specific mode of paired statistical variant analysis provided by Mutect2. Otherwise, we used the unpaired analysis mode.

*Variant annotation and advanced filtering.* Next, we applied a multi-stage filter hierarchy to optimize the specificity for our somatic mutation calls. After basic artefact filtering steps, we annotated discovered variants with all available transcript contexts and their protein level consequences using TransVar v2.4.1 (12) and the NCBI RefSeq gene models (13). In case of multiple RefSeq transcripts per gene, we annotated each variant on protein level with its strongest possible biological consequence. For mutation overview plots, we later selected the first principal transcript of the respective gene according to the APPRIS database (14). Additionally, we annotated variants with confirmed somatic mutations according to the Catalog of Somatic Mutations in Cancer (COSMIC v85) (15), the NCBI database of common human variants (≥5% in any of the five large populations from dbSNP build 151(16)), and NCBI ClinVar (version 2018-04) (17) using vcfanno v0.3.0 (18). To filter FFPE specific artifacts, we fitted the read orientation model of GATK (LearnReadOrientationModel) that flags all variants associated with a significant bias between reads for the forward and reverse strands. As countermeasure for alignment artifacts caused by high sequence homology, e.g., from pseudogenes, we additionally filtered variants utilizing the GATK FilterAlignmentArtifacts model (Supplementary Table S2). Driver gene mutations were predicted by the MutSig2CV algorithm and are described in Supplementary Table S4.

*Called somatic mutations.* All basic and advanced filter steps based on variant statistics from Mutect2, GATK, and all annotations are listed in their applied processing order in Supplementary Table S5, including detailed mutation counts and percentages remaining after each step. Overall, our filter hierarchy called 0.05% of all discovered variants somatic mutations in FL, which corresponds to 1.35 mutations per sample and Mb on average.

For the comparison of mutations in defined subcohorts (Supplementary Table S3) Wilcoxon rank sum test was used to determine significant differences (p<0.05) in the respective groups. For correction of multiple hypothesis testing the Benjamini and Hochberg (BH) method was performed, indicating prescribed error threshold (False discovery rate *FDR*: q) of q< 0.1 as significant.

Additional tools and software utilized

For various analysis tasks in the sequencing pipeline, we used bedtools 2.26.0 (19), the Integrative Genomics Viewer (IGV v2.10.2) (20), the Picard toolkit (https://broadinstitute.github.io/picard/), and SAMtools (21). For analysis pipeline orchestration including parallel remote analysis jobs on high-performance clusters as well as for most visualizations including oncoplots, we used MATLAB® (versions R2021a-R2022a, The MathWorks® Inc., Natick, Massachusetts, USA). We used Microsoft Excel (versions 2016-365) for collecting clinical metadata and presenting results. R (version 4.X, R Foundation for Statistical Computing, Vienna, Austria), Python (version 2.7-3.X, Python Software Foundation, Wilmington, Delaware, USA), and GNU parallel (22) were used for running various tools or for local parallelization. TMB was computed and visualized together with TCGA data using maftools v2.12.05 (23). Needle plots of mutation profiles were created using ProteinPaint (24).

Detecting somatic copy number alterations (SCNA) using the OncoScan CNV Assay

Briefly, 40-80ng of DNA were used for analysis. Conversion of raw data into CN data was processed by the Chromosome Analysis Suite (ChAS) software (v4.0, Thermo Fisher Scientific, Waltham, Massachusetts, USA). For single-sample analysis, raw data were transformed into OSCHP files for further evaluation in ChAS displaying losses, gains and copy number neutral loss of heterozygosity (CNN-LOHs) for each chromosome. To call CNAs using ChAS, the prerequisites included a size of at least 100kbp and a coverage of at least 15 markers, as previously described (25). Additionally, the thresholds for the log2 ratio was set to 0.30 and -0.25 for gains and losses, respectively. For CNN-LOHs, a minimal size of 5000 kbp was determined. To illustrate the most frequent gains and losses for the cohort, the multi sample viewer (MSV, v 1.0.0.144), was used. For allele-specific copy number segmentation at sample level, we applied ASCAT v2.4.3 (26). Resulting copy numbers for genes are summarized in Supplementary, Table S6. ASCAT also estimated sample ploidy and purity, i.e., the cell fraction originating from aberrant tumor cells opposed to non-aberrant bystander cells. Two FFPE samples were excluded due to ASCAT quality control. The remaining 147 were available for SCNA discovery. Recurrent SCNA at the cohort level were identified and statistically evaluated using GISTIC 2.0 (27) (Supplementary Table S7). Significant SCNA were defined by cohort frequency ≥5% and qG2.0<0.1. False discovery rates (*FDR*) were computed using the Benjamini-Hochberg method. For significance, we used a prescribed error threshold of q< 0.1.

The Oncoscan CNV Assay data of 122 sFL samples was used for descriptive analysis, comparing SCNA of lFL and sFL with ChAS v4.0. Recurrent SCNA occurring in ≥15% were comparatively analyzed in lFL and sFL using Fisher´s exact test and Benjamini-Hochberg correction for multiple testing.

*Estimating the cancer cell fraction and clonality.* Mutation clonality was determined by integrating targeted sequencing data with SNP results. We used available SNP array data to estimate tumor purity, ploidy, and copy numbers, and combined this information with variant allele frequencies from the targeted sequencing data to compute the cancer cell fractions (CCF) carrying specific mutations. CCF was estimated using the formula

f_CCF=f_VAF/f_purity ((1-f_purity )∙n_(CN,normal)+f_purity 〖∙n〗_(CN,tumor) )

which takes into account variant allele frequency f_VAF_, tumor purity f_purity_, normal cell copy number n_CN,normal_=2 (assuming diploid cells), and ASCAT-determined tumor cell copy number n_CN,tumor_. Clonal variants were defined as those with a threshold of f_CCF_ ≥ 0.9. The estimated CCF and all identified mutations can be found in Supplementary Table S5 (refer to the column group 'Variant def./call quality/details' for tumor).

Data availability

The WES and SCNA data generated in this study have been deposited in the European Genome-phenome Archive (EGA) under study accession EGAS00001006927 (<https://ega-archive.org/studies/EGAS00001006927>). All methods, tools, resources and software used in this study are summarized in Supplementary Table S8.

Evaluation of the *BCL2* and *BCL6* translocation status by Fluorescence in situ hybridization (FISH) and delta-PCR

For FISH analysis, FFPE tissue samples were either available on tissue microarray (TMA) or as whole tissue sections. FISH was performed on 4µM tissue sections that were hybridized with the Vysis LSI *BCL2* and the Vysis LSI *BCL6* dual color break-apart probe (Abbott Molecular, Chicago, Illinois, USA), as previously described (28, 29). Cases of insufficient quality or lacking sections that were not evaluable by FISH were examined for the *BCL2* translocation by delta-PCR assay (30) with multiplex primers for major and minor breakpoint regions of the *IGH* and *BCL2* genes. For some cases of the institutional archives, chromosome banding data were available determining the *BCL2* translocation status for those samples.

SCNA validation by fluorescence in-situ hybridization (FISH)

Target-specific fluorescent-labelled probes were prepared using clones of bacterial artificial chromosomes (BACs) RP11-673F7 (*IKZF1,* 7p12.2), RP11-933C1 (*FCRL5,* 1q23.1), RP11-629A20 (*ETS1*, 11q24.3) and RP11-953P9 (*ADAM32*, 8p11.22). After cultivation of the specific BAC clones (Thermo Fisher Scientific, Waltham, Massachusetts, USA), midiprep for the purification of grown BAC-DNA was performed after protocol using the PureYield Plasmid Midiprep System Kit (Promega, Madison, Wisconsin, USA). To validate the purified DNAs, a PCR with locus-specific primers was performed and finally labelled by random priming using the Invitrogen BioPrime DNA labeling system Kit (Thermo Fisher Scientific, Waltham, Massachusetts, USA). Hybridization of selected cases for SCNA validation was performed using the manufactured FISH probes as previously described (29). As control probes Vysis CEP1 DNA Probe Kit and Vysis CEP7 DNA Probe Kit (Abbott Molecular, Chicago, Illinois, USA) were used.

Investigation of target-specific gene expression (GE) by RT-PCR and usage of GE data from previously published data sets

RT-PCR was conducted using 20ng cDNA, previously synthetized by the AB High Capacity cDNA RT Kit (Thermo Fisher Scientific, Waltham, Massachusetts, USA) using 1000ng of RNA. TaqMan™ GE assays were used for *ADAM32* (Hs00991935_m1), *ETS1* (Hs00428293_m1), *FCRL5* (Hs01070204_m1), *IKZF1* (Hs00958474_m1) and *POLR2B* (Hs00265358_m1) as reference. All samples were analyzed in triplicates on an AB 7500 TaqMan instrument (Thermo Fisher Scientific, Waltham, Massachusetts, USA) and quantified using the standard curve or delta-delta Ct method. For analyses of GE profiles, subgroups (e.g. lFL with or without *KIR3DL1* mutations) were compared according their underlying GE, as assessed previously (1). GraphPad Prism 9.4.1 (GraphPad Software, La Jolla, CA) was used for statistical analysis applying by Mann-Whitney U test to determine the significance (in general, two sided; one sided in case of a directed hypothesis) of the association between two variables.

**Supplementary Tables Legends**

**Supplementary Table S1**: Cohort overview and samples metadata

**Supplementary Table S2**: WES variant filtering hierarchy

**Supplementary Table S3**: Comparisons of mutation frequencies in subcohorts

**Supplementary Table S4**: Driver gene predictions by MutSig2CV

**Supplementary Table S5**: Called somatic mutations

**Supplementary Table S6:** Copy numbers for genes based on ASCAT segments for lFL

**Supplementary Table S7:** Called SCNA for lFL

**Supplementary Table S8:** Methods, tools, resources and software used in this study

| **Method/Tool/Software** | **Version** | **Available at** | **Notes** |
| --- | --- | --- | --- |
| HISAT2 | 2.2.1 | <http://daehwankimlab.github.io/hisat2/download> | alignment method |
| Genome Analysis Toolkit (GATK) / Mutect | 4.1.2.0 | <https://github.com/broadinstitute/gatk/releases> | GATK contains Mutect 2 for variant discovery |
| TransVar | 2.4.1 | <https://github.com/zwdzwd/transvar> | variant annotator (installed via pip) |
| Chromosome Analysis Suite (ChAS) | 4.3 | <https://www.thermofisher.com/chas> | software suite from SNP array manufracturer |
| ASCAT | 2.4.3 | <https://github.com/Crick-CancerGenomics/ascat> | copy number segmentation and sample purity |
| GISTIC | 2.0 | <https://broadinstitute.github.io/gistic2/> | cohort level SNCA analysis (Matlab runtime required) |
| Integrated Genomics Viewer | 2.10.2 | <http://software.broadinstitute.org/software/igv/download> | variant plots in context of their measured reads |
| GNU parallel | 20161222 | <https://www.gnu.org/software/parallel/> | local parallelization of jobs capsuled as bash scripts |
| samtools | 1.9 | <http://www.htslib.org> | sequence file related tasks (manual QC, resorting, indexing) |
| bedtools | 2.26.0 | <https://bedtools.readthedocs.io> | QCs |
| Picard tools | 2.25.0 | <https://broadinstitute.github.io/picard/> | sequence file related tasks (manual QC, resorting, indexing, fastq extraction) |
| vcfanno | 0.3.0 | <https://github.com/brentp/vcfanno/releases> | quickly combine variant annotation sources |
| Trim Galore! | 0.6.6 | <https://github.com/FelixKrueger/TrimGalore> | automation of cutadapt and FastQC iterations |
| cutadapt | 3.2 | <https://cutadapt.readthedocs.io/en/stable/> | preprocessing of sequencing data: cut adapter sequences |
| FastQC | 0.11.9 | <http://www.bioinformatics.babraham.ac.uk/projects/fastqc> | QC of sequencing measurements |
| **Resources/Databases** | **Version** | **Available at** | **Notes** |
| COSMIC | 85 | <https://cancer.sanger.ac.uk/cosmic> | resource database of known somatic mutations |
| NCBI ClinVar | 20180429 | <https://www.ncbi.nlm.nih.gov/clinvar/> | resource database of variants with known clinical significance |
| gnomAD/ExAC | based on v2 | provided via GATK resource pack (af-only-gnomad.hg38.ensemble.vcf.gz); created from gnomAD by <https://github.com/broadinstitute/gatk/blob/master/scripts/mutect2_wdl/mutect_resources.wdl> | resource database of known population germline variants, originally based on ExAC |
| NCBI Common Human Variants | “common_all.vcf.gz” from 20180418 | <https://www.ncbi.nlm.nih.gov/variation/docs/human_variation_vcf> | resource database of common human variants, part of dbSNP build 151 |
| NCBI RefSeq gene models | 20190227 | provided via TransVar download; file name hg38.refseq.gff.gz.transvardb; downloaded 20190227 | gene models used for advanced variant annotation, e.g. in the codon frame context |
| APPRIS | 20200122 | <https://appris.bioinfo.cnio.es/#/downloads> | principal isoforms/transcripts of genes |
| Human Reference Genome | GRCh38 | <http://daehwankimlab.github.io/hisat2/download/#h-sapiens> | besides the reference genome, this resource contains indexes for sequence alignment via HISAT2; the identical reference genome was utilized for all downstream analyses |
| **IDEs and runtimes** | **Version** | **Available at** | **Notes** |
| MATLAB | R2022a | <https://www.mathworks.com/pricing-licensing.html?prodcode=ML&intendeduse=edu> | general purpose analysis software suite (license required) |
| Python | 2.7 and 3.6 | <https://www.python.org/> | general purpose language and runtime (needed by TransVar) |
| R | 3.6.3 and 4.1.0 | <https://www.r-project.org/> | Statistics analysis language and runtime (needed by ASCAT) |

**Supplementary Figure Legends**

**Supplementary Figure S1: Comparative analyses of lFL and sFL.**

Schematic overview of FL samples analyzed by whole exome sequencing (WES) and genome-wide somatic copy number alterations (SCNA), separated into localized FL (lFL) and systemic FL (sFL), and with respect to their underlying *BCL2* translocation status (*BCL2* translocation-positive: *BCL2*+; *BCL2* translocation-negative: *BCL2*-)

**Supplementary Figure S2: Distribution of mutations in FL.**

Tumor mutational burden of solid and hematologic malignancies compared with the present FL cohort showed an intermediate TMB (A). The average number of mutations per sample was 83. The number of mutations per sample did not differ significantly in lFL and sFL (B).

**Supplementary Figure S3: Mutational landscape in sFL.**

All called non-synonymous and synonymous mutations in significant genes according to MutSig2CV v3.11 (qM2CV < 0.1, cohort frequency ≥ 12.5%) and in non-significant biologically-relevant genes (e.g. *BCL2*) are color-coded and shown for sample per column, ranked by cohort frequency. Samples are ordered by waterfall sorting based on binary gene mutation status. The bar graph on the left shows the ratio of non-synonymous (blue) and synonymous (green) mutations per gene. At the top, the TMB per sample (mutations/sample/Mb) is depicted. On the right, occurring types of mutation and q values (M2CV) are shown per gene.

**Supplementary Figure S4: Comparative analysis of SCNA in lFL stage I and II.**

Frequency of SCNA in the entire cohort of localized FL (lFL), separated into FL stage I and stage II. Copy number gains along the genome are depicted in red (above); copy number losses are illustrated in blue (below).

**Supplementary Figure S5: Somatic copy number alterations in the lFL cohort.**

GISTIC v2.0 defined copy number gains (red) are visualized for each chromosome, arm-level alterations on the left, and focal lesions on the right. Insignificant lesions (qG2.0 > 0.1) are shaded in gray. Selected potential driver genes within significant focal lesions are highlighted with corresponding FDR q-values (A). Copy number deletions (blue) are correspondingly shown for each chromosome (B).

**Supplementary Figure S6: Identification of novel focal SCNA and putative target genes in known regions of gain.**

Significant SCNA affecting chromosome 1q23.1 by gain (red), 8p11.22 by loss (blue) and chromosome 11q24.3 (red). Validation of newly identified genes in the significant region of gains by Fluorescence in situ hybridization (FISH) in 1q23.1 (*FCRL5*, A), of losses in 8p11.22 (*ADAM32*, B) and of gains in 11q24.3 (*ETS1,* E), as well as the consequence of the respective SCNA on mRNA expression for *FCRL5* (B), *ADAM32* (D) and *ETS1* (F)*.* For the comparison of GE in samples with or without gain/loss by Mann-Whitney U‑test was applied to determine significant differences (* p<0.05).

**Supplementary Figure S7: Comparing mutational profiles of lFL and sFL reveals slight differences.**

Differences in mutational spectrum of *KMT2D* (A) and *CREBBP* (B) in localized FL (lFL) and systemic FL (sFL), showing the occurence of splicing site mutations (yellow) in lFL exclusively. Mutations affecting the *KIR3DL1* gene were solely observed in lFL with predominantly missense mutations affecting the immunoglobulin domain of the protein (C). Compared to lFL samples without *KIR3DL1* mutation (wildtype: WT), FL samples with *KIR3DL1* mutations (MUT) showed significantly increased mRNA expression of *CD4*, *ITK* and *SH2D1A* when compared with patient samples lacking *KIR3DL1* mutations (D). For the comparison of GE in samples with or without *KIR3DL1* mutation by Mann-Whitney U‑test was applied to determine significant differences (* p<0.05).

**Supplementary Figure S8: Comparative analysis of SCNA in lFL with or without *BCL2* translocations.**

Frequency of SCNA in the entire cohort of lFL, separated into lFL with *BCL2* translocation (*BCL2*+) and without *BCL2* translocation (*BCL2*-). Copy number gains along the genome are depicted in red (above); copy number losses are illustrated in blue (below).

**Supplementary Figure S9: Comparative cluster analysis of *BCL2* translocation-negative lFL and sFL (analog (31))**

Comparative analysis of *BCL2* translocation-negative lFL and sFL considering the respective frequency of *BCL6* translocations, as well as mutations in *CREBBP* and *STAT6*.

**Supplementary Figure S1**

**
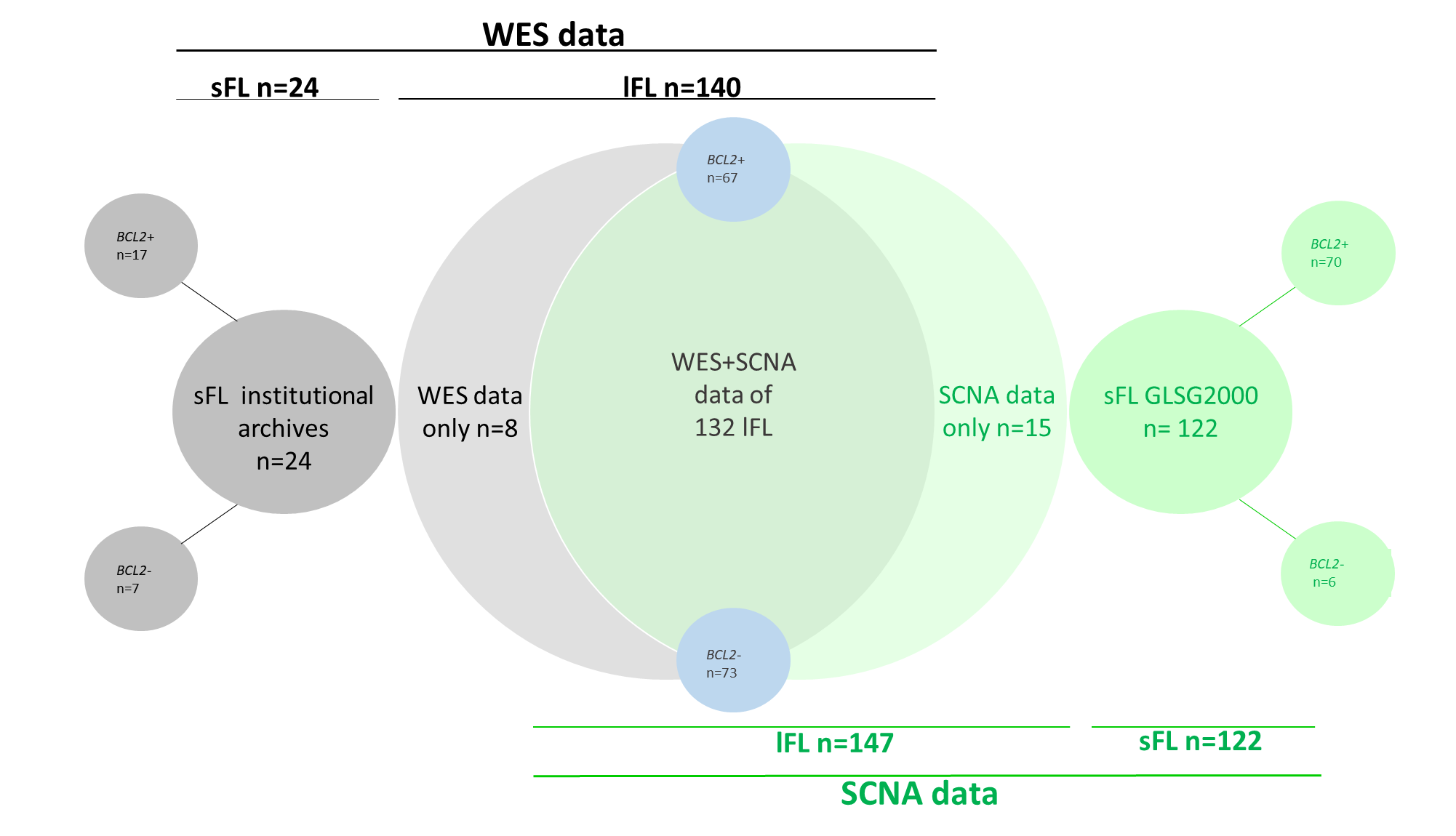
**

**Supplementary Figure S2**


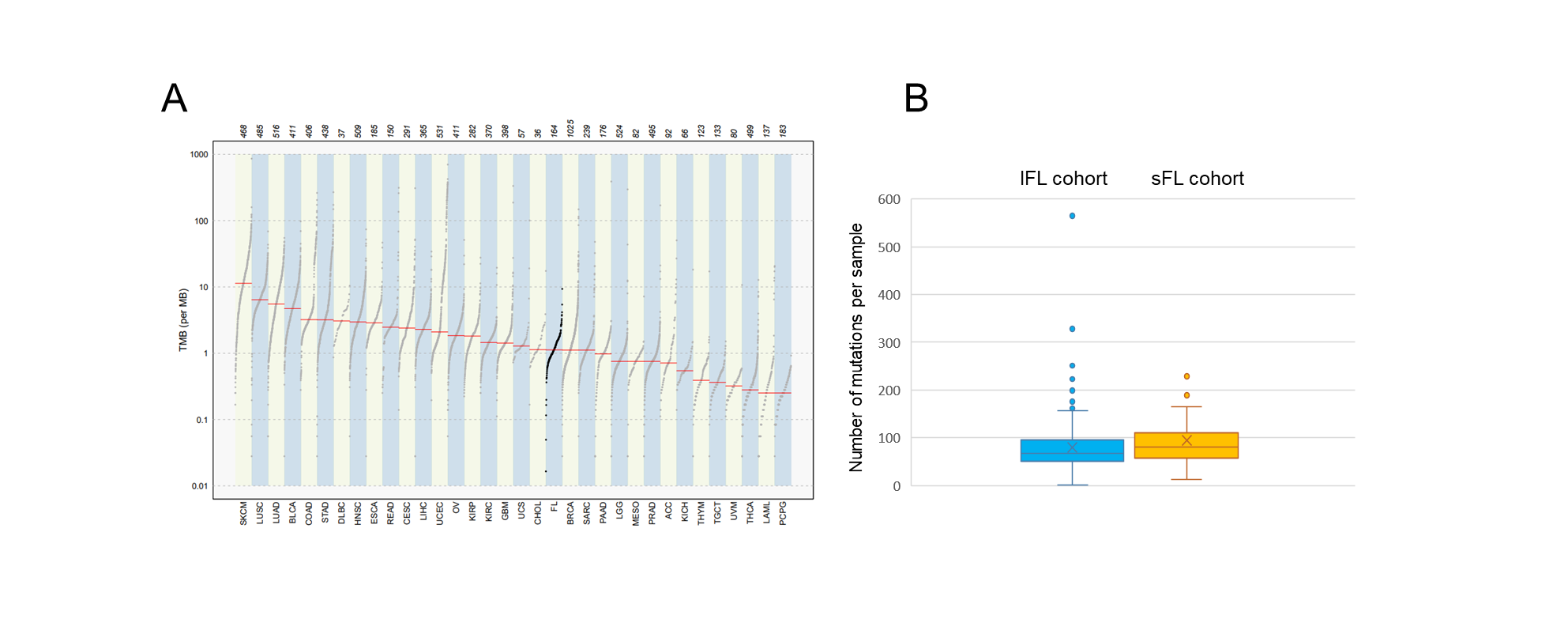


**Supplementary Figure S3**


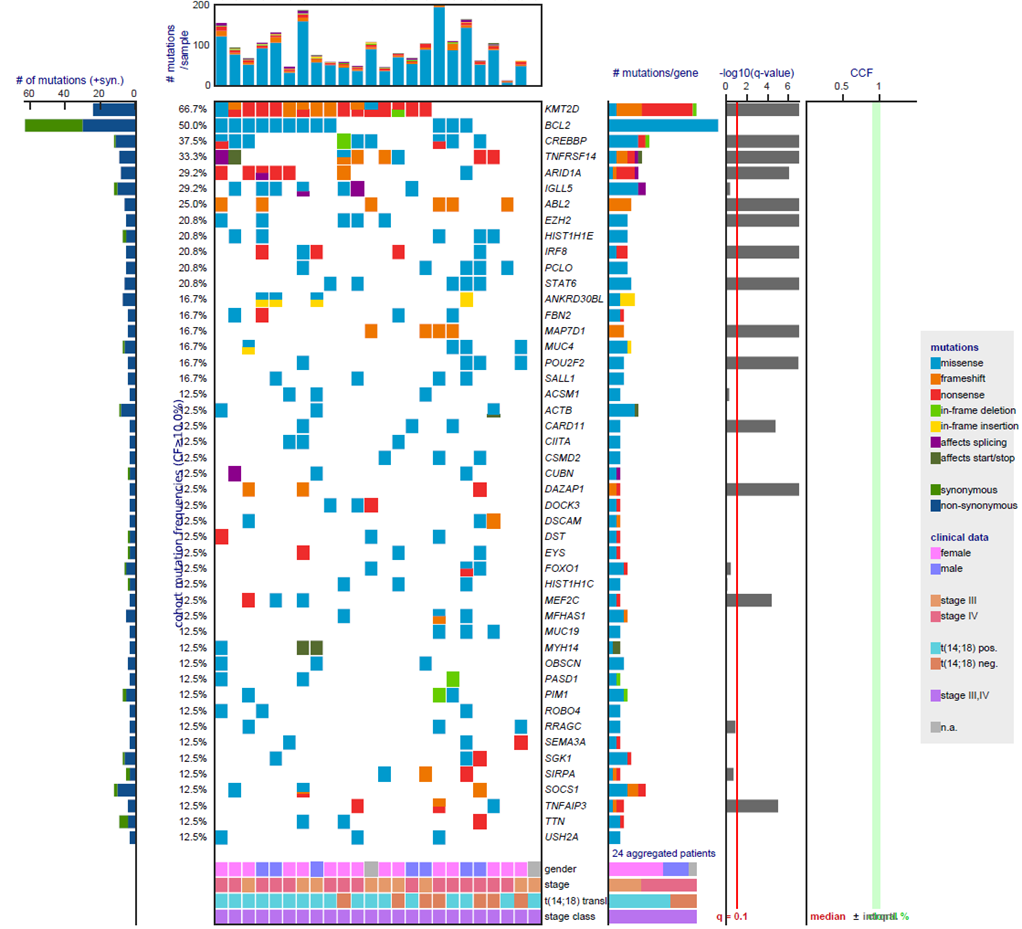


**Supplementary Figure S4**


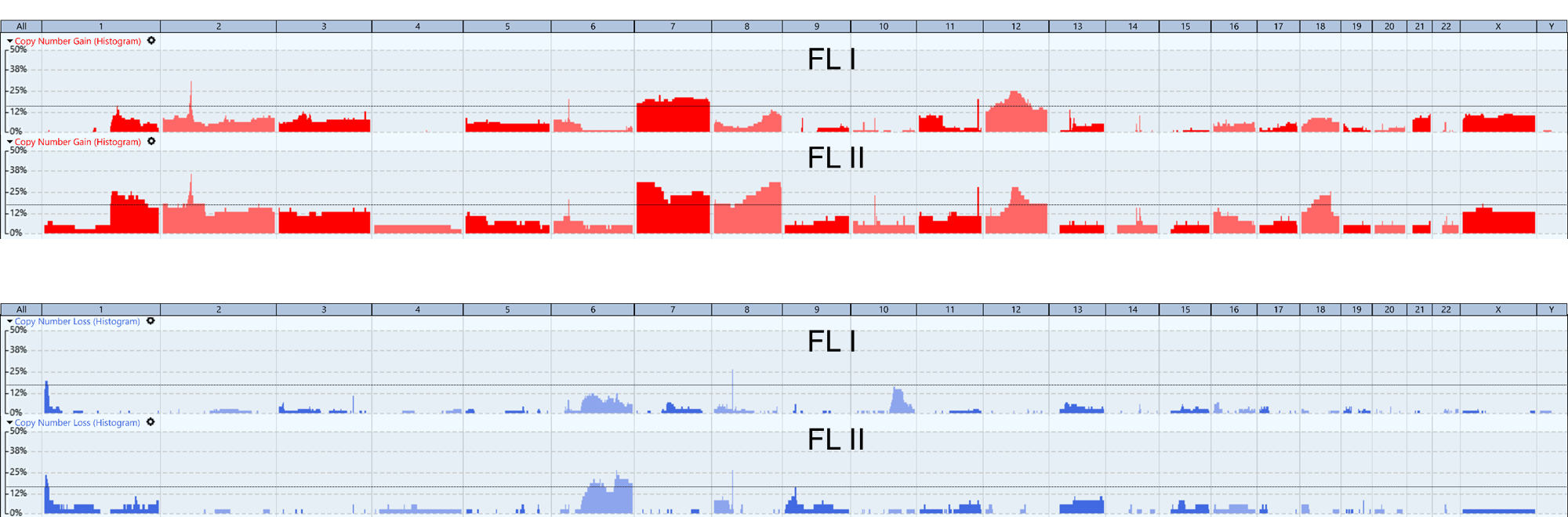


**Supplementary Figure S5**


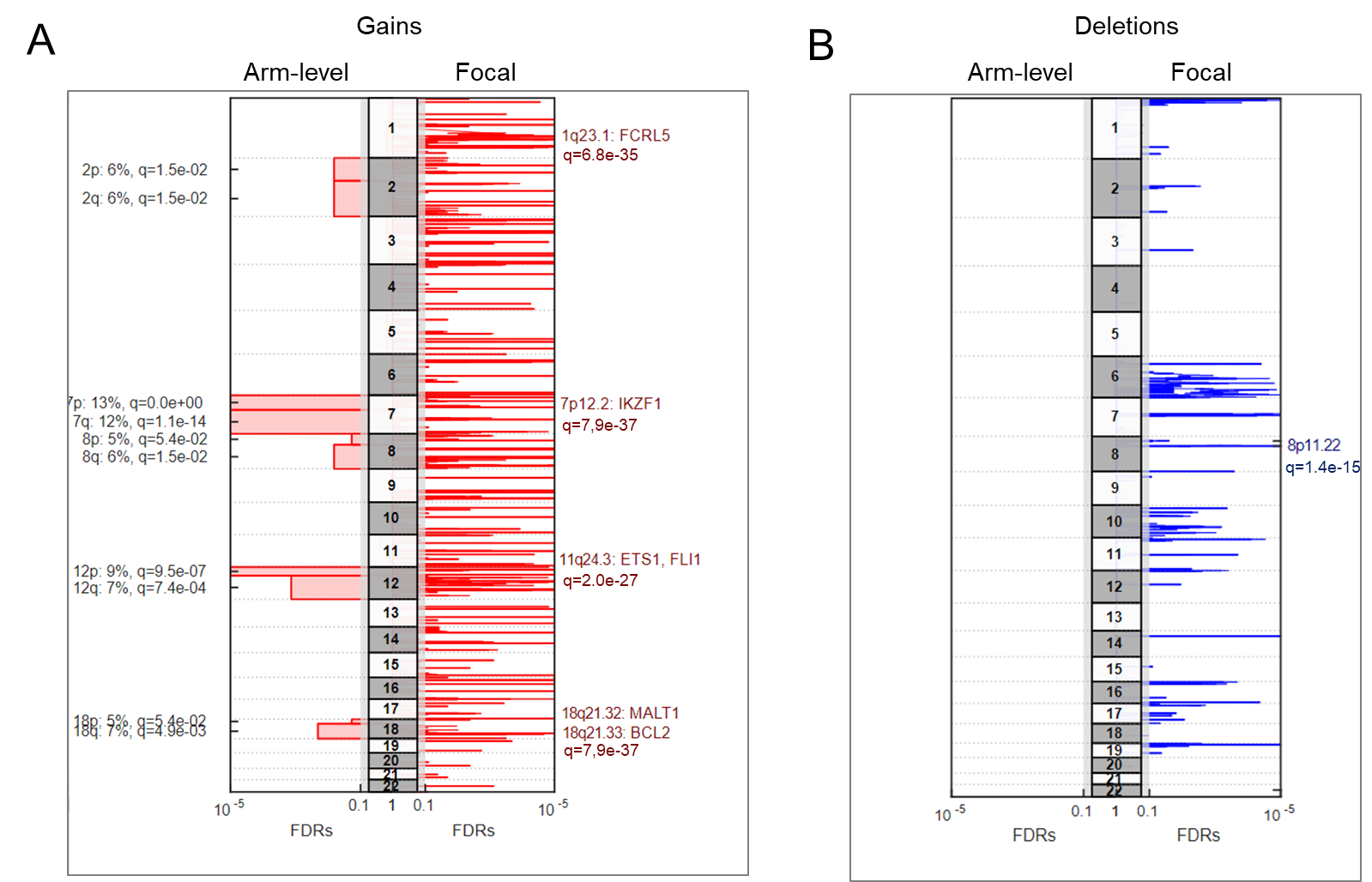


**Supplementary Figure S6**


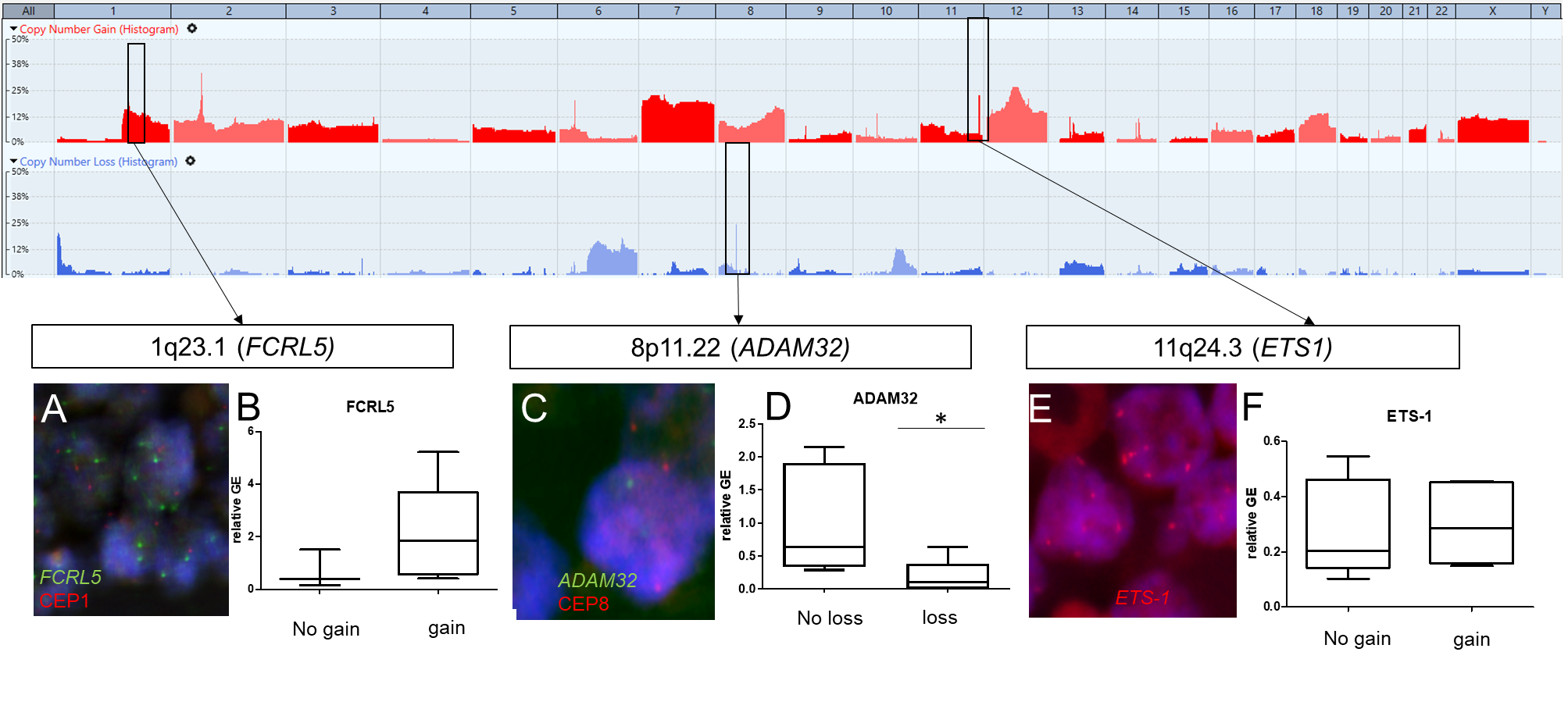


**Supplementary Figure S7**


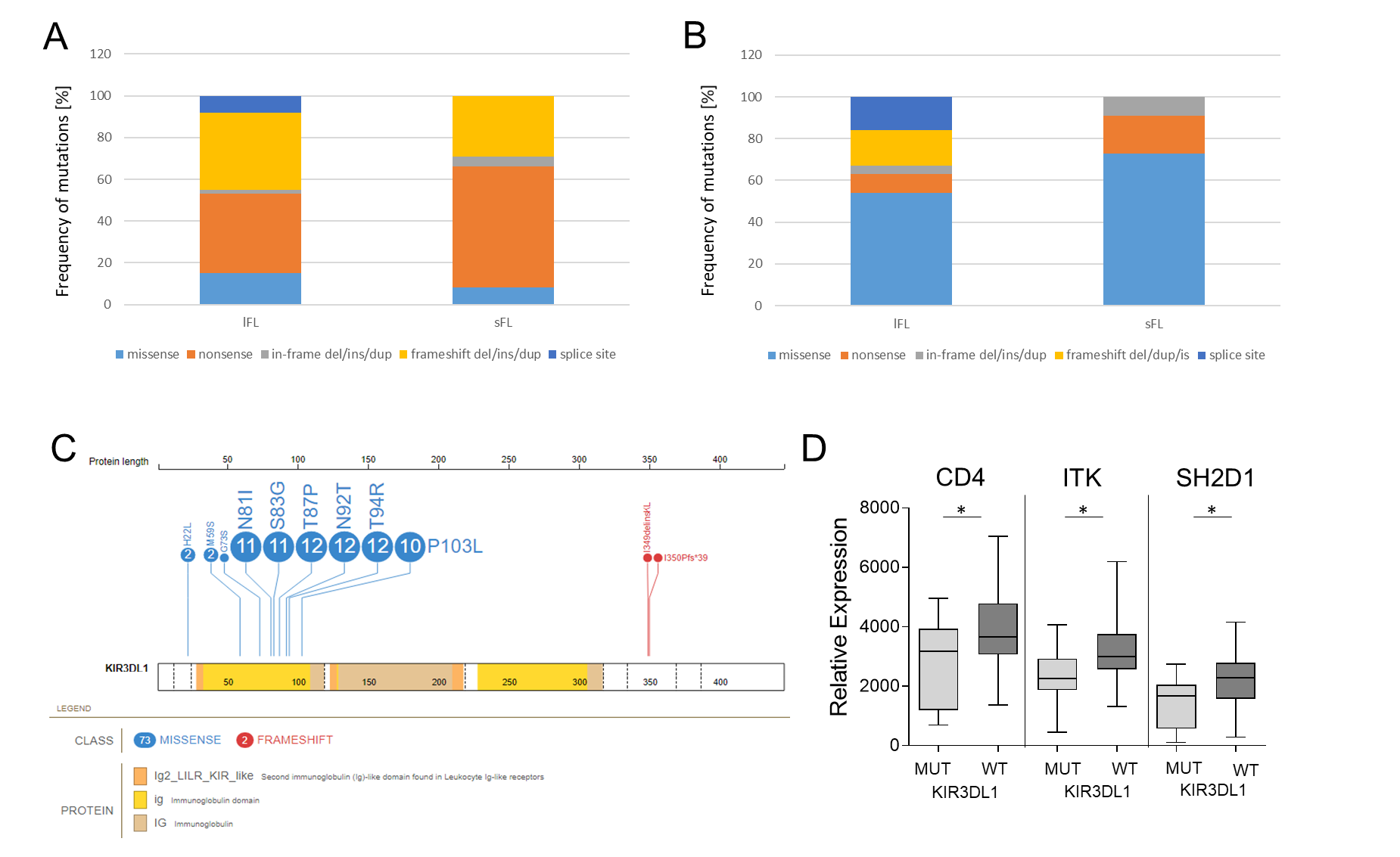


**Supplementary Figure S8**


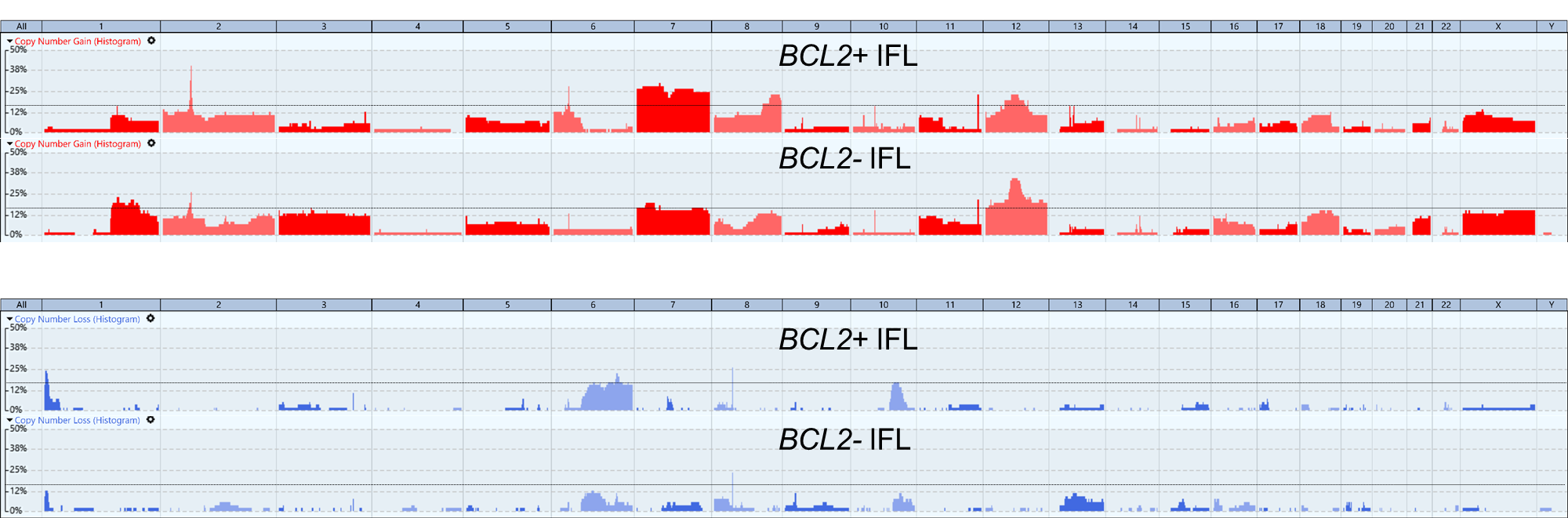


**Supplementary Figure S9**


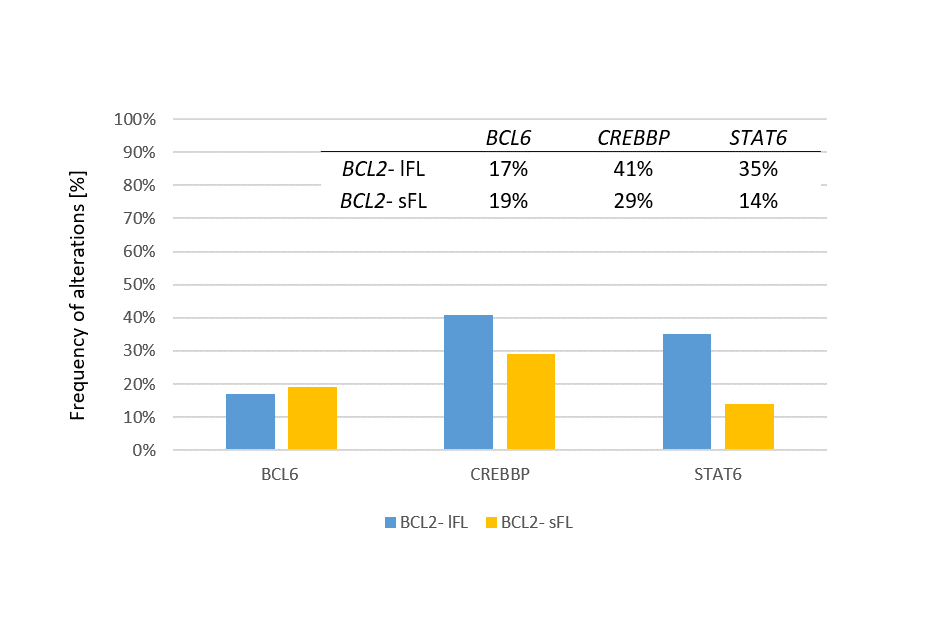


**German Lymphoma Alliance (GLA)**

Vindi Jurinovic^6^, Eva Hoster^6^, Oliver Weigert^6^, Klaus Herfarth^8^, Wolfram Klapper^13^, Martin Dreyling^6^, Georg Lenz^4^, Andreas Rosenwald^5^, German Ott^1,2^

^1^Department of Clinical Pathology, Robert-Bosch Hospital, Stuttgart, Germany

^2^Dr. Margarete Fischer-Bosch Institute of Clinical Pharmacology, Stuttgart, Germany,

^4^Department of Medicine A, Department of Hematology, Oncology and Pneumology, University Hospital Münster, Münster, Germany,

^5^Institute of Pathology, University of Würzburg and Comprehensive Cancer Center Main, Würzburg, Germany,

^6^Department of Medicine III, University Hospital, LMU Munich, Munich, Germany

^8^Department of Radiation Oncology, University of Heidelberg, Heidelberg, Germany,

^13^Institute of Pathology, Hematopathology Section and Lymph Node Registry, University Hospital Schleswig-Holstein, Campus Kiel, Kiel, Germany

**References**

1. Staiger AM, Hoster E, Jurinovic V, Winter S, Leich E, Kalla C, et al. Localized- and advanced-stage follicular lymphomas differ in their gene expression profiles. *Blood* 2020; **135**: 181–190.

2. Staiger AM, Ott MM, Parmentier S, Rosenwald A, Ott G, Horn H, et al. Allele-specific PCR is a powerful tool for the detection of the MYD88 L265P mutation in diffuse large B cell lymphoma and decalcified bone marrow samples. *British journal of haematology* 2015; **171**: 145–148.

3. Martin M. Cutadapt removes adapter sequences from high-throughput sequencing reads. *EMBnet.journal* 2011; **17**: 10–12.

4. Krueger F. Trim Galore! is a wrapper script to automate quality and adapter trimming.: at https://www.bioinformatics.babraham.ac.uk/projects/trim_galore/ 2012.

5. Andrews S. FastQC: a quality control tool for high throughput sequence data.: at http://www.bioinformatics.babraham.ac.uk/projects/fastqc 2010.

6. Kim D, Langmead B, Salzberg SL. HISAT: a fast spliced aligner with low memory requirements. *Nature methods* 2015; **12**: 357–360.

7. Schneider VA, Graves-Lindsay T, Howe K, Bouk N, Chen H-C, Kitts PA, et al. Evaluation of GRCh38 and de novo haploid genome assemblies demonstrates the enduring quality of the reference assembly. *Genome research* 2017; **27**: 849–864.

8. Bergmann EA, Chen B-J, Arora K, Vacic V, Zody MC. Conpair: concordance and contamination estimator for matched tumor-normal pairs. *Bioinformatics (Oxford, England)* 2016; **32**: 3196–3198.

9. Lee S, Lee S, Ouellette S, Park W-Y, Lee EA, Park PJ. NGSCheckMate: software for validating sample identity in next-generation sequencing studies within and across data types. *Nucleic acids research* 2017; **45**: e103.

10. McKenna A, Hanna M, Banks E, Sivachenko A, Cibulskis K, Kernytsky A, et al. The Genome Analysis Toolkit: a MapReduce framework for analyzing next-generation DNA sequencing data. *Genome research* 2010; **20**: 1297–1303.

11. Lek M, Karczewski KJ, Minikel EV, Samocha KE, Banks E, Fennell T, et al. Analysis of protein-coding genetic variation in 60,706 humans. *Nature* 2016; **536**: 285–291.

12. Zhou W, Chen T, Chong Z, Rohrdanz MA, Melott JM, Wakefield C, et al. TransVar: a multilevel variant annotator for precision genomics. *Nature methods* 2015; **12**: 1002–1003.

13. O'Leary NA, Wright MW, Brister JR, Ciufo S, Haddad D, McVeigh R, et al. Reference sequence (RefSeq) database at NCBI: current status, taxonomic expansion, and functional annotation. *Nucleic acids research* 2016; **44**: D733-45.

14. Rodriguez JM, Maietta P, Ezkurdia I, Pietrelli A, Wesselink J-J, Lopez G, et al. APPRIS: annotation of principal and alternative splice isoforms. *Nucleic acids research* 2013; **41**: D110-7.

15. Tate JG, Bamford S, Jubb HC, Sondka Z, Beare DM, Bindal N, et al. COSMIC: the Catalogue Of Somatic Mutations In Cancer. *Nucleic acids research* 2019; **47**: D941-D947.

16. Sherry ST, Ward MH, Kholodov M, Baker J, Phan L, Smigielski EM, et al. dbSNP: the NCBI database of genetic variation. *Nucleic acids research* 2001; **29**: 308–311.

17. Landrum MJ, Lee JM, Benson M, Brown GR, Chao C, Chitipiralla S, et al. ClinVar: improving access to variant interpretations and supporting evidence. *Nucleic acids research* 2018; **46**: D1062-D1067.

18. Pedersen BS, Layer RM, Quinlan AR. Vcfanno: fast, flexible annotation of genetic variants. *Genome biology* 2016; **17**: 118.

19. Quinlan AR, Hall IM. BEDTools: a flexible suite of utilities for comparing genomic features. *Bioinformatics (Oxford, England)* 2010; **26**: 841–842.

20. Thorvaldsdóttir H, Robinson JT, Mesirov JP. Integrative Genomics Viewer (IGV): high-performance genomics data visualization and exploration. *Briefings in bioinformatics* 2013; **14**: 178–192.

21. Danecek P, Bonfield JK, Liddle J, Marshall J, Ohan V, Pollard MO, et al. Twelve years of SAMtools and BCFtools. *GigaScience* 2021; **10**.

22. Tange O. GNU Parallel - The Command-Line Power Tool. *Login USENIX Mag.* 2011; **36**: 42–47.

23. Mayakonda A, Lin D-C, Assenov Y, Plass C, Koeffler HP. Maftools: efficient and comprehensive analysis of somatic variants in cancer. *Genome research* 2018; **28**: 1747–1756.

24. Zhou X, Edmonson MN, Wilkinson MR, Patel A, Wu G, Liu Y, et al. Exploring genomic alteration in pediatric cancer using ProteinPaint. *Nature genetics* 2016; **48**: 4–6.

25. Wagener R, Seufert J, Raimondi F, Bens S, Kleinheinz K, Nagel I, et al. The mutational landscape of Burkitt-like lymphoma with 11q aberration is distinct from that of Burkitt lymphoma. *Blood* 2019; **133**: 962–966.

26. van Loo P, Nordgard SH, Lingjærde OC, Russnes HG, Rye IH, Sun W, et al. Allele-specific copy number analysis of tumors. *Proceedings of the National Academy of Sciences of the United States of America* 2010; **107**: 16910–16915.

27. Mermel CH, Schumacher SE, Hill B, Meyerson ML, Beroukhim R, Getz G. GISTIC2.0 facilitates sensitive and confident localization of the targets of focal somatic copy-number alteration in human cancers. *Genome biology* 2011; **12**: R41.

28. Horn H, Jurinovic V, Leich E, Kalmbach S, Bausinger J, Staiger AM, et al. Molecular Cytogenetic Profiling Reveals Similarities and Differences Between Localized Nodal and Systemic Follicular Lymphomas. *HemaSphere* 2022; **6**: e767.

29. Horn H, Bausinger J, Staiger AM, Sohn M, Schmelter C, Gruber K, et al. Numerical and structural genomic aberrations are reliably detectable in tissue microarrays of formalin-fixed paraffin-embedded tumor samples by fluorescence in-situ hybridization. *PloS one* 2014; **9**: e95047.

30. Lin M-T, Tseng L-H, Rich RG, Hafez MJ, Harada S, Murphy KM, et al. Δ-PCR, A Simple Method to Detect Translocations and Insertion/Deletion Mutations. *The Journal of molecular diagnostics JMD* 2011; **13**: 85–92.

31. Los-de Vries GT, Stevens WBC, van Dijk E, Langois-Jacques C, Clear AJ, Stathi P, et al. Genomic and microenvironmental landscape of stage I follicular lymphoma, compared with stage III/IV. *Blood advances* 2022; **6**: 5482–5493.
